# The Effect of Electronic-Cigarette Vaping on Cardiac Function and Angiogenesis in Mice

**DOI:** 10.1038/s41598-019-40847-5

**Published:** 2019-03-11

**Authors:** Huilin Shi, Xiaoming Fan, Austin Horton, Steven T. Haller, David J. Kennedy, Isaac T. Schiefer, Lance Dworkin, Christopher J. Cooper, Jiang Tian

**Affiliations:** 1 Department of Medicine, University of Toledo College of Medicine, Toledo, OH 43614 USA; 2 Department of Medicinal and Biological Chemistry, College of Pharmacy and Pharmaceutical Sciences, University of Toledo, Toledo, OH 43614 USA

## Abstract

The rapid increase in use of electronic-cigarettes (e-cigarettes), especially among youth, raises the urgency for regulating bodies to make informed decisions, guidance, and policy on these products. This study evaluated cardiac function in an experimental model following exposure to e-cigarettes. We subjected C57BL/6 mice to e-cigarette vaping for 2-weeks, and cardiac function was assessed using echocardiography. Cardiac tissues were collected at the end of e-cigarette exposure for pathological analysis. The experimental data showed that e-cigarette vaping (3 h/day for 14 days) had no significant effect on cardiac contractility as measured by ejection fraction. However, it significantly increased angiogenesis in mouse heart tissue. We found that e-cigarette exposure increased the endothelial cell marker CD31 and CD34 to approximately 2 fold (p < 0.05) in heart tissue from female mice and about 150% (p < 0.05) in male mice. E-cigarette vaping also caused slower weight gain compared to mice exposed to room air. In addition, short-term e-cigarette exposure slightly increased collagen content in heart tissue but did not result in significant tissue fibrosis. These results suggest that short-term exposure to e-cigarettes has no acute effect on cardiac contractile function or tissue fibrosis, but it increases cardiac angiogenesis.

## Introduction

Tobacco smoking has been demonstrated to be a major risk factor for heart failure and is associated with the morbidity in heart failure patients^1–6^, while the effect of electronic cigarette (e-cigarette) use is largely unknown. E-cigarettes are interpreted in many societies as a safer alternative compared to combustible cigarettes (c-cigarettes) despite the fact that there is no sufficient evidence regarding e-cigarette safety and efficacy for replacing c-cigarettes^7–11^. There are very few studies investigated the potential effect of e-cigarettes on cardiac function, and most of these studies are cell culture-based or small size clinical studies. Patented in 2003 and extensively promoted in the U.S. for the past decade, e-cigarettes are estimated to become a $10 billion dollar industry^12^. The marketing of e-cigarettes as a healthy alternative to c-cigarette smoking is associated with increased use of e-cigarettes among younger adolescents and current smokers who believe that e-cigarettes are not harmful^13–17^.

Experimentally, some early studies showed that e-cigarette liquid or vapor were less toxic compared to c-cigarettes in cultured cardiac myocytes and endothelial cells^18–21^. A recent study using longitudinal within-subjects observational method showed that switching from c-cigarettes to e-cigarettes substantially reduced several carcinogens and toxicants such as the metabolites of 1,3-butadiene, benzene and acrylonitrile, while nicotine exposure remains unchanged^22^. However, in other studies, it was found that significant amounts of formaldehyde and acetaldehyde in e-cigarette vapor, and at higher temperature, trace amount of acetone and acrolein were detectable^23^, suggesting some shared toxicity between e-cigarettes and c-cigarettes. It was reported that e-cigarettes and associated flavoring agents may produce harmful effects in stem cells and gingival fibroblasts by generating aldehydes/carbonyls from e-cigarette vapor, resulting in protein carbonylation and DNA damage, as well as cellular senescence^24^. Habitual e-cigarette use was found to shift cardiac autonomic balance toward sympathetic predominance and increased oxidative stress, which are associated with increased cardiovascular risk^25^. These results raised questions regarding the safety of e-cigarette use and its beneficial effect as a substitute for c-cigarettes.

Our recent studies suggest that chronic exposure to e-cigarette vaping disrupts airway barrier function, induces tissue fibrosis in the heart and kidney, and causes systemic inflammation in mice^26^. In the current study, we investigated the acute effect of short-term exposure to e-cigarettes on cardiac function and tissue injury in mice.

## Results

### The effect of e-cigarette vaping on mouse cardiac function and bodyweight gain

**Figure 1:**
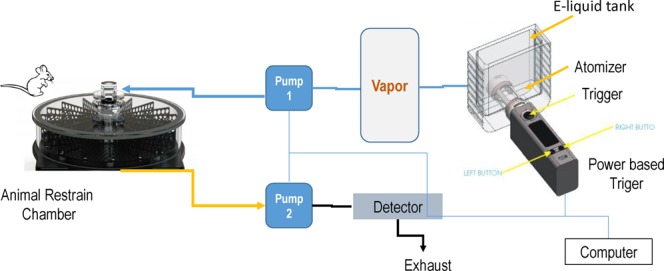
Schematic presentation of the InExpose e-cigarette vaping system.

**Figure 2:**
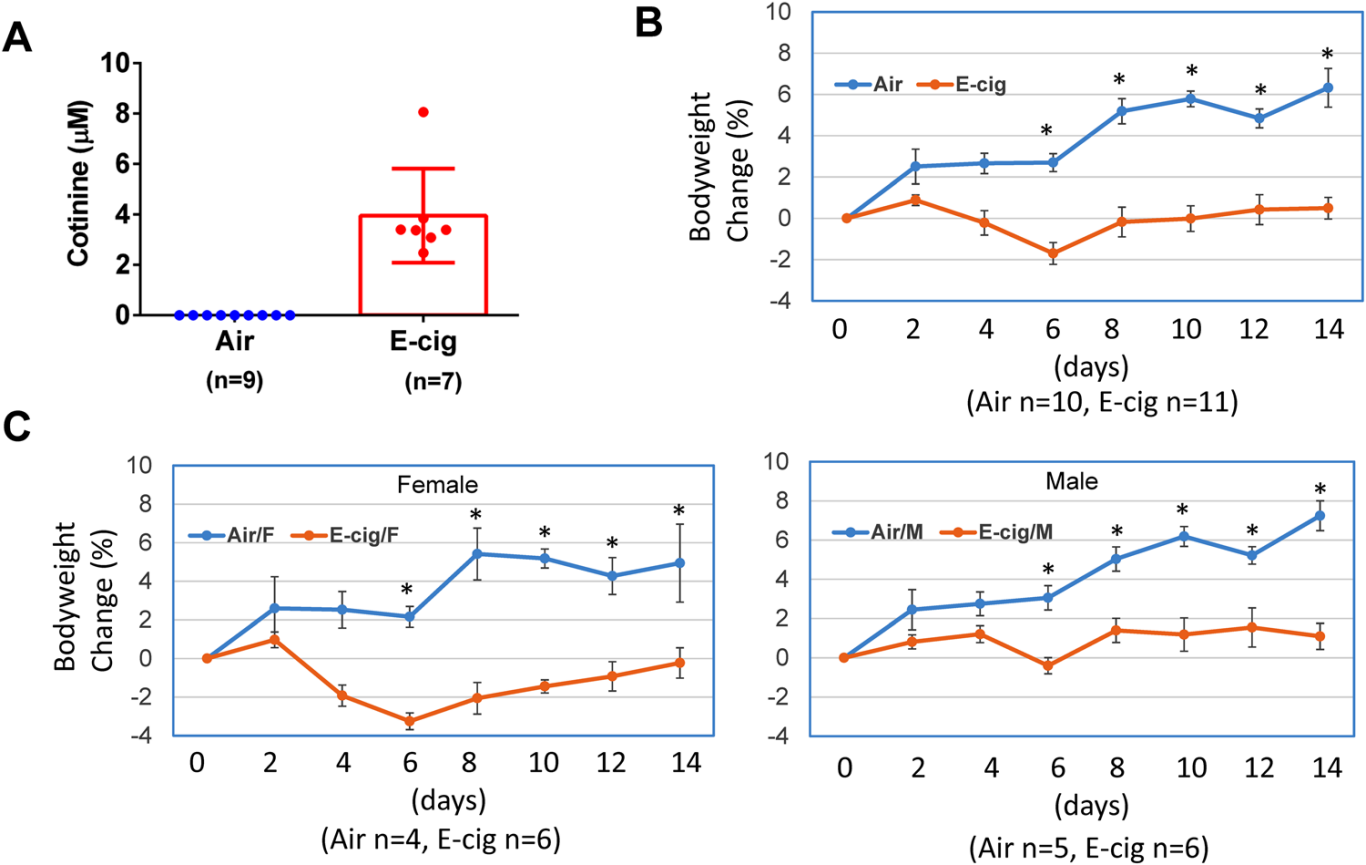
E-cigarette vaping inhibits mouse bodyweight gain. Mice were exposed to air or e-cigarette vapor for 14 days and bodyweight was measured every two days. The percent change was calculated based on the bodyweight at baseline (one day before exposure). Blood was drawn from heart at the end of last e-cigarette exposure. (**A**) Plasma concentration of cotinine. (**B**) Percentage change of bodyweight including both male and female mice. (**C**) Percentage change of bodyweight in male and female mice, respectively. *Indicates p < 0.05 at the same time point.

**Figure 3:**
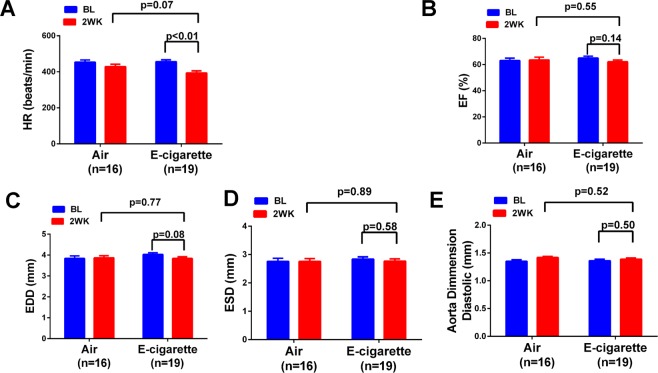
Effect of e-cigarette vaping on mouse cardiac function. Echocardiographic measurement was performed at baseline and at the end of the 2^nd^ week. The heart rate (**A**), ejection fraction (**B**) end diastolic dimension (EDD) (**C**), end systolic dimension (ESD) (**D**) and aorta dimension (**E**) were measured and compared between air controls and those subjected to e-cigarette exposure.

**Figure 4:**
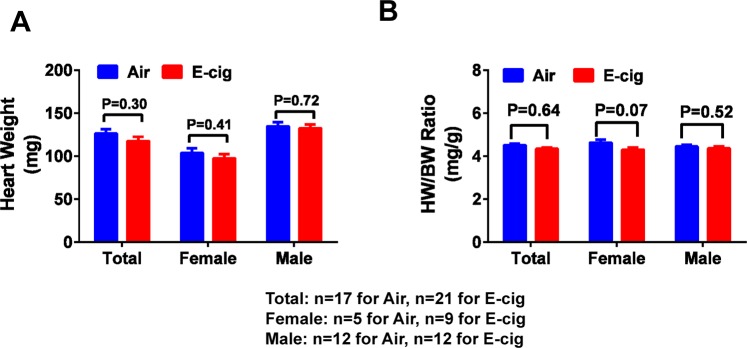
Heart weight and heart weight/body weight ratio. (**A**) The animal heart was collected after euthanization at the end of e-cigarette exposure, and heart weight was measured after removing the aorta and other tissue debris. (**B**) Heart weight/Body weight (HW/BW) ratio was calculated using the baseline bodyweight.

We have previously demonstrated that c-cigarette smoking worsens cardiac and renal function in humans and in animal models^27,28^. To study the effect of e-cigarette smoking on the cardiac functional change, we performed echocardiographic measurements on animals exposed to e-cigarette vapor. The e-cigarette liquid was made of propylene glycol and glycerin at 1:1 ratio and contains 24 mg/ml nicotine. E-cigarette vapor was generated using InExposure cigarette smoking system from SCIREQ as shown in Fig. 1 as described in Methods section. The body weight of each mouse was measured every two days during the experiment and the percentage body weight change was calculated. To determine the nicotine exposure level in these animals, we measured the plasma concentration of cotinine, a major metabolite of nicotine. As shown in Fig. 2A, the cotinine concentration was about 3.95 ± 0.70 µM in e-cigarette-exposed mice, while it was not detectable in air-exposed mice. In body weight measurement, we found that e-cigarette vaping significantly inhibited the bodyweight gain (Fig. 2B). This reduction in bodyweight gain was significant in both male and female mice compared to controls (Fig. 2C). Echocardiographic data showed that e-cigarette vaping for 2 weeks significantly decreased the heart rate compared to the baseline, whereas the heart rate in air control groups had no significant differences (Fig. 3A). E-cigarette vapor caused a modest decrease in ejection fraction compared to baseline but was not statistically significant (Fig. 3B). There was no significant change in left ventricle chamber size and aorta dimension after e-cigarette exposure (Fig. 3C–E). The heart weight in mice exposed to e-cigarette vapor (particularly the female mice) were lower compared to that in the air control group (103.6 ± 5.53 vs 97.61 ± 4.61 mg in female; 134.85 ± 4.6 vs 132.60 ± 4.12 in male), but had no statistical significance (Fig. 4A). The heart weigh/body weight (HW/BW) ratio showed a similar trend (Fig. 4B).

### Short-term exposure to e-cigarettes had no significant effect on cardiac fibrosis

**Figure 5:**
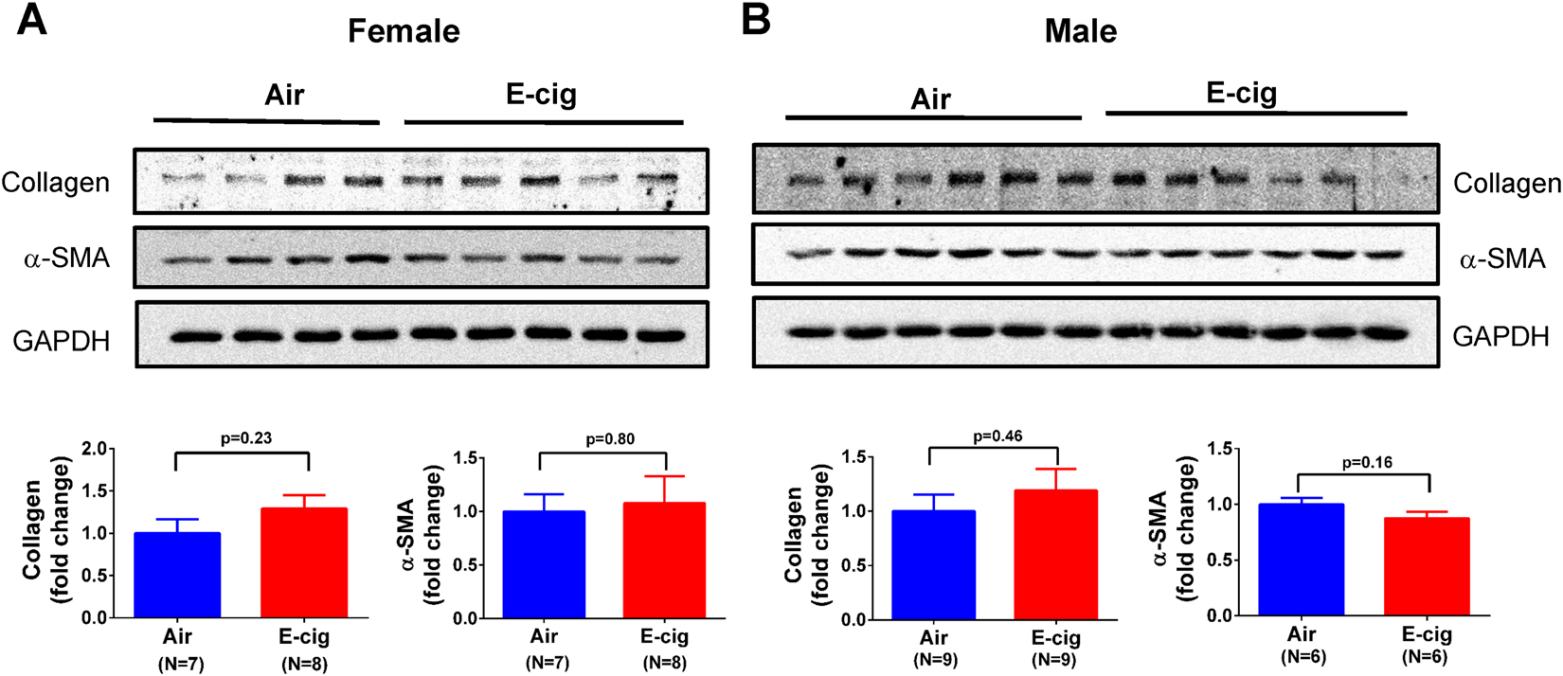
Effect of e-cigarette exposure on collagen and α-SMA expression in mouse cardiac tissue. Heart tissue homogenates from female (**A**) and male (**B**) mice were analyzed using Western blot to probe the collagen I and α-SMA expression. The upper panels were representative Western blot images and the lower panels showed the quantification data.

**Figure 6:**
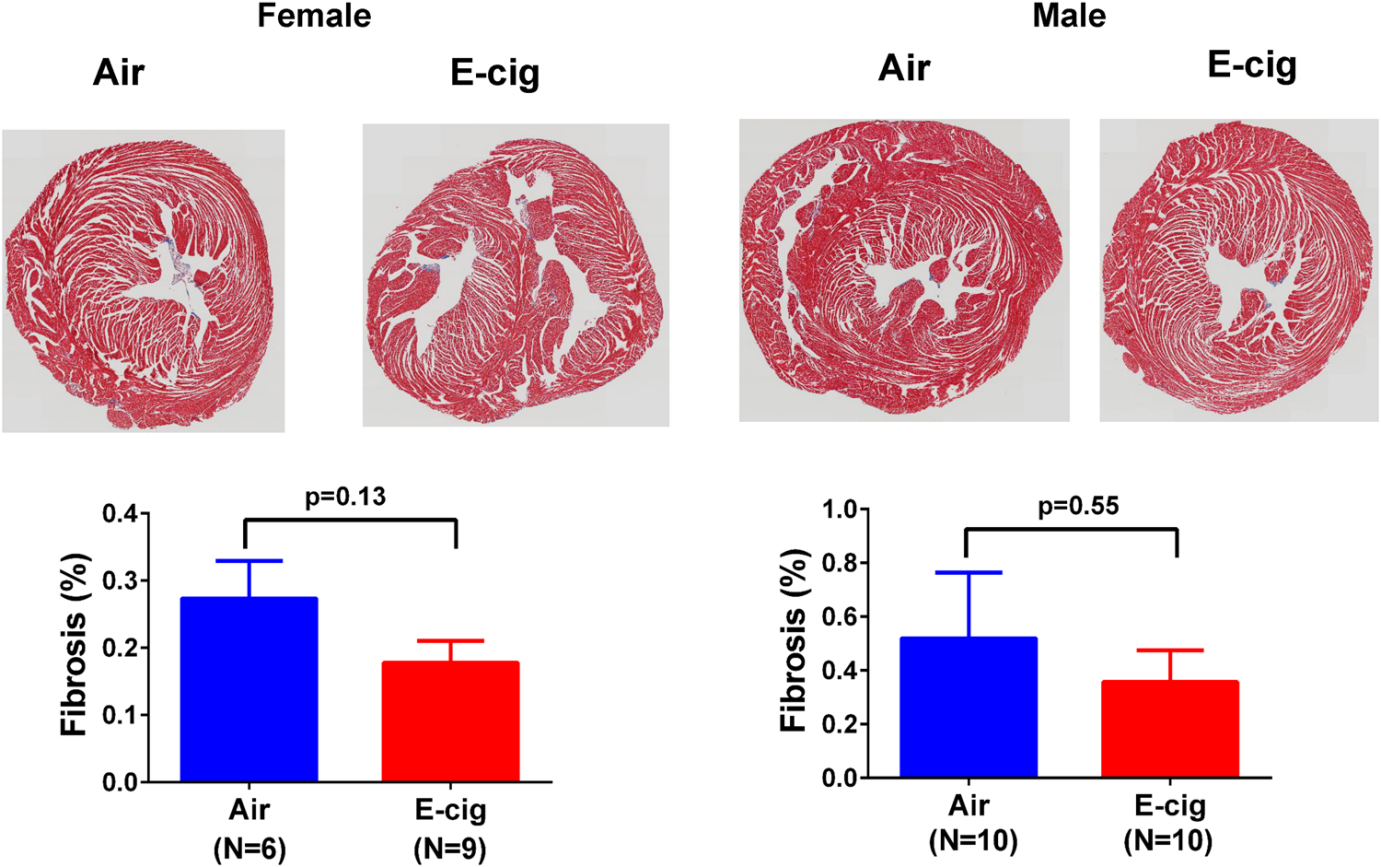
Effect of e-cigarette exposure on cardiac tissue fibrosis. Formaldehyde fixed and paraffin-embedded heart tissue was cut into 4 µm sections onto a specimen. Trichrome staining was performed and analyzed as described in Method section. Whole slide images were taken by an Olympus whole-slide scanner with a 20x lens.

To examine the potential pathological effect of e-cigarettes on cardiac tissue, we first analyzed the fibrosis markers, collagen and α-SMA, in the heart tissue homogenates from these animals using Western blot. As shown in Fig. 5, the result showed that e-cigarette vapor slightly increased collagen I protein expression compared to the air control group in both female and male mice, but had no statistical significance. To a similar extent, e-cigarette exposure had no significant effect on the expression of α-SMA in these animals. We then performed Trichrome staining for tissue fibrosis in heart sections and found that there was no significant change in Trichrome staining between the air control and e-cigarette-exposed mice (Fig. 6), suggesting that short-term exposure to e-cigarette vapor does not cause significant tissue fibrosis.

### Exposure to e-cigarettes increase heart tissue angiogenesis

**Figure 7:**
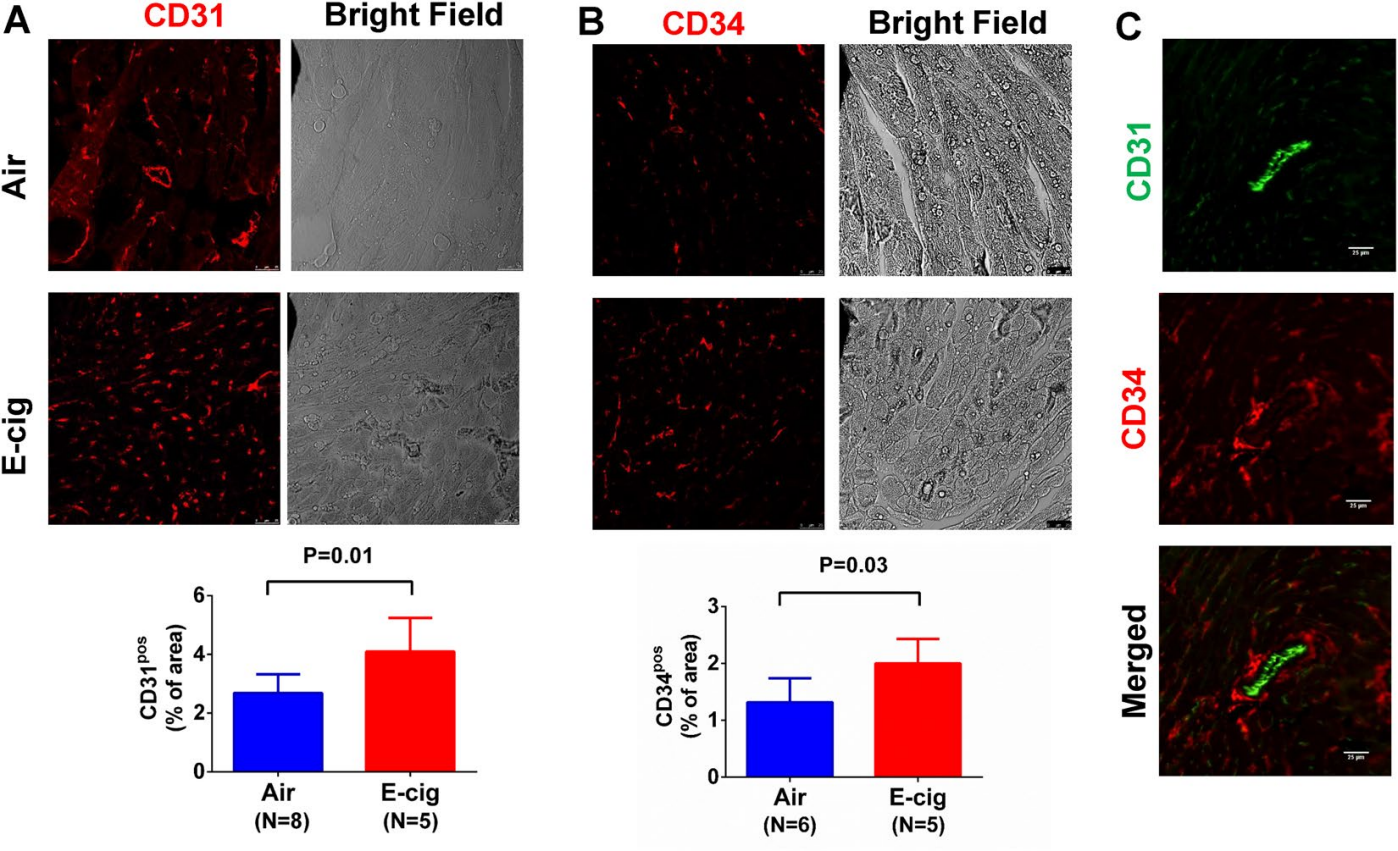
E-cigarette increases capillary density. (**A**) Heart tissue sections were immunostained with anti-CD31 antibody and Alexa-594 conjugated secondary anti-rabbit antibody. (**B**) Heart tissue was immunostained with anti-CD34 antibody and Alexa-594 conjugated secondary antibody. (**C**) Co-immunostaining of CD31 (green) and CD34 (red) in heart tissue sections. Five fluorescent Images from each slide were taken using a Leica Confocal microscope. The percentage of CD31 or CD34 positive area was analyzed using FiJi ImageJ software. Scale bar is 25 µm.

**Figure 8:**
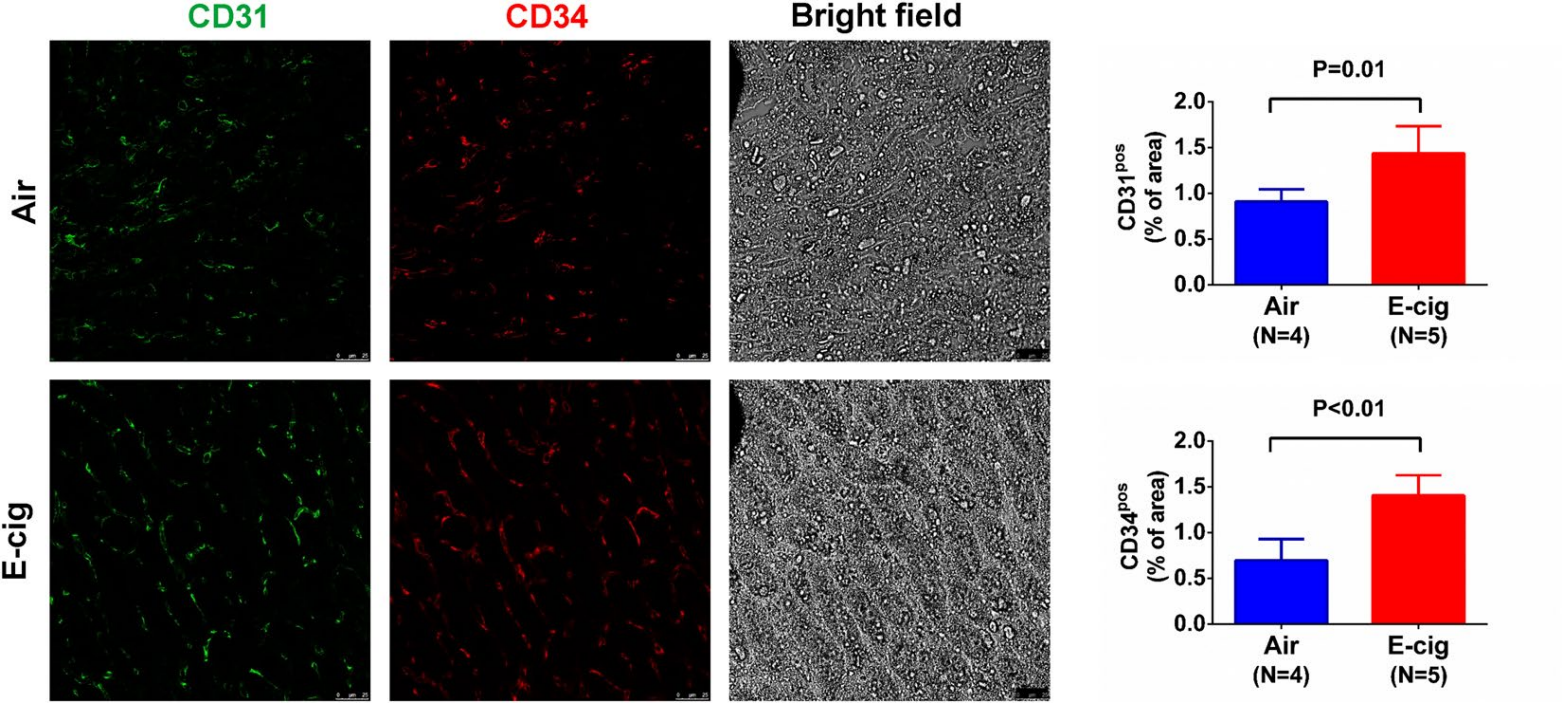
E-cigarette increase CD31 and CD34 expression in kidney tissue. Kidney tissue from air control (Air) and e-cigarette (E-cig) were co-immunostained with CD31 (green) and CD34 (red) with anti-CD31 and anti-CD34 antibodies. Five fluorescent images from each slide were taken using a Leica confocal microscope. The percentage of CD31 or CD34 positive area was analyzed using FiJi ImageJ software. Scale bar is 25 μm.

**Figure 9:**
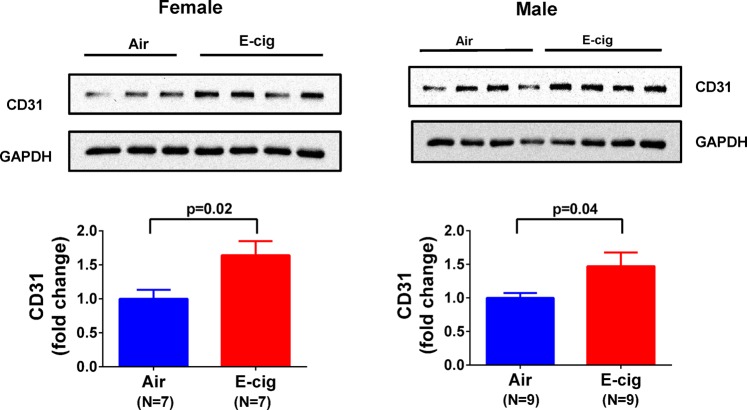
Effect of e-cigarettes on CD31 expression in cardiac tissue. Heart tissue homogenates from female or male mice were separated on a SDS-PAGE gel and the expression of CD31 was analyzed using Western blot with anti-CD31.

**Figure 10:**
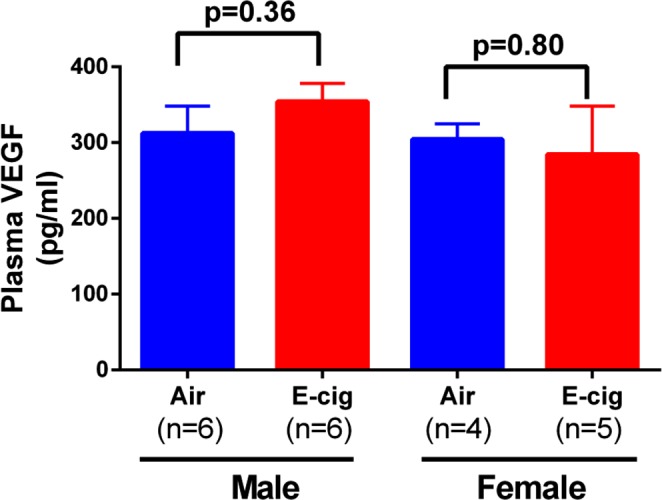
E-cigarette vaping on plasma VEGF levels. Blood samples were collected at the last day of experiment immediately after e-cigarette or air exposure. Plasma was prepared by centrifugation and plasma levels of VEGF were measured using a commercial ELISA kit.

It is known that nicotine increases angiogenesis but its effect could be masked by other chemicals in cigarette smoking. To test the effect of e-cigarettes on heart tissue angiogenesis, we measured CD31, an endothelial and angiogenesis marker, in these animals after e-cigarette exposure. As shown in Fig. 7A, the capillary density measured by CD31 positive immunostaining showed that e-cigarette exposure increased CD31 positive areas compared to air exposure. We also immunostained CD34, a commonly used angiogenesis marker, in the cardiac tissue. The result showed a similar trend as CD31 (Fig. 7B), suggesting that e-cigarette exposure increases cardiac tissue angiogenesis. However, when CD31 and CD34 were co-immunostained, we found that the CD31 was mostly located at the lumen side of a vessel while CD34 was located mostly at the outside of the vessel (Fig. 7C). In kidney tissue sections, CD31 and CD34 were also significantly increased after e-cigarette exposure (Fig. 8). To further quantify the CD31 expression, we performed Western blot and showed significant increase in CD31 expression in heart tissue from mice exposed to e-cigarettes (Fig. 9). To understand how e-cigarettes increases angiogenesis and capillary density, we measured the VEGF level in blood samples using an ELISA kit. As shown in Fig. 10, there was no significant differences in the VEGF levels between e-cigarette-exposed mice and air controls.

## Discussion

Our current research showed that short term exposure to e-cigarette vaping does not have significant effect on cardiac contractility and geometric properties, but it significantly increases the angiogenesis represented by capillary density in mouse heart tissue. It is known that combustible cigarette smoking in general inhibits tissue angiogenesis despite the fact that nicotine alone actually stimulates angiogenesis^29^. The inhibition effect of smoking on angiogenesis is considered detrimental especially to pregnant woman and fetus development^30^. Our current data showed that exposure to e-cigarette vapor containing nicotine have a significant increase in CD31 and CD34, suggesting an increase in angiogenesis. However, the physiological effect of the increased angiogenesis is not clear at this point. Increased angiogenesis is beneficial in the condition of myocardial infarction, but the effect of e-cigarettes on myocardial infarction need to be further studied in animal models and in humans. Angiogenesis could also promote tumor growth and increase chances of atherogenesis. In the current study, we did not find an increase in plasma VEGF levels after e-cigarette exposure as other studies have found in nicotine-induced angiogenesis^29^, which may be due to the time point when we collected the blood samples. Therefore, the mechanisms that e-cigarette vaping causes increased angiogenesis need to be further studied. In addition, the current study did not use any commercial e-cigarette or e-liquid because there are too many different brands with different formula in the market. Our rational is first to examine the effect of a standard formula with essential components before testing the role of additive components.

Our data also showed that heart rate was decreased after two weeks of e-cigarette vaping in mice. This is consistent with the observation in our recent publication demonstrating that smoking causes a decrease in heart rate in animals^28^. However, the heart rate in both studies was measured under anesthesia, which may not reflect the real heart rate in conscious conditions. In addition, the short term exposure of e-cigarettes only causes modest changes in heart function and geometric properties. Unlike the long term chronic exposure which causes multiple organ fibrosis as we recently published^26^, short term e-cigarette vaping resulted in no significant increase in heart tissue fibrosis.

Our study also found that exposure to e-cigarettes caused significant inhibition of bodyweight gain in these animals. However, since our initial experimental design was not aimed to study the bodyweight change, we did not measure the food and water consumption in these animals. Therefore, it is not clear whether the effect of e-cigarettes on bodyweight gain is through decreased food intake or through other mechanisms.

In summary, our current study demonstrates that short-term exposure to e-cigarettes may increase tissue angiogenesis, which represents a novel indication for the pathophysiological effects of acute e-cigarette exposure that needs to be further elucidated.

## Materials and Methods

### Animal

Animal experiments were conducted in accordance with the National Institutes of Health, Guide for the Care and Use of Laboratory Animals under protocols (IACUC# 108714) approved by the Institutional Animal Care and Use Committee at the University of Toledo. Adult male and female C57BL/6 mice at 2–3 months of age were used for this study. All mice were reared under a 12 h dark/light cycle, fed with standard chow, and provided with water *ad libitum*. These conditions were utilized for the entire duration of the experiment.

### E-cigarette Exposure Protocol

A whole body smoking exposure system from SCIREQ was used for the experiment. This specialized computer-operated machinery generates e-cigarette vapor using a controlled power supply (as shown in Fig. 1) and is housed in an exhaust hood. Male and female mice were held in the restraint chamber connected to the e-cigarette vaping system. The whole system is placed in an exhaust hood. Control mice were held in a separate chamber that exposed to room air only. The e-liquid consists of 50% propylene glycol, 50% glycerin, and 24 mg/ml nicotine as described previously^31^. The e-liquid filled in a chamber with an atomizer was vaporized at a frequency of 1 puff per minute and duration of 10 seconds per puff. Generated vapor was delivered to the mouse restraint chamber using an air pump and subsequently cleared out by a second pump. Both pumps as well as the vaping trigger were controlled by the InExposure computer software. Total exposure time was 3 hours per day with a 10 min break every hour for 14 consecutive days. Mice were acclimated for 30 min in the restraint chamber before the first exposure to e-cigarette vapor.

### Blood Samples and Organ Collection

Blood was collected from the heart with a syringe after animals were euthanized with xylazine at the last day of e-cigarette exposure. The plasma fraction was obtained by centrifugation and stored at −80 °C. Following blood collection, the heart was collected and weighed. One half of the heart was flash frozen and stored at −80 °C for use in biochemical assessments of cardiac toxicity. The other half of the heart was fixed in 4% formaldehyde solution and processed for morphometric analysis of tissue injury as we previously published^32–34^.

### Cardiovascular Function Assessments

Echocardiographic measurements was performed once at baseline (one day prior to e-cigarette exposure) and repeated at 14 days after e-cigarette exposure. The left ventricle diastolic dimension, systolic dimension, wall thickness, and heart rate were measured using an Acuson Sequoia C512 (Siemens) ultrasound machine as previously described^35–37^. Briefly, animals were anesthetized with 2% isoflurane delivered on 100% Oxygen. The chest area was treated with Nair to remove the hair. An ultrasonic probe was used to assess the left ventricle size. The end diastolic dimension (EDD) and end systolic dimension (ESD) were measured using M-mode on short axis position. The diastolic volume and systolic volume were estimated using the following formula: Diastolic Volume = 4/3*π*(EDD*1/2)^3^; Systolic Volume = 4/3*π*(ESD*1/2)^3^, assuming that the left ventricle shape is spherical. Ejection fraction (EF%) was calculated as: EF% = (Diastolic volume-Systolic Volume)/Diastolic volume * 100%. The aorta dimension was measured by positioning the probe to the aorta area in long axis.

### Western Blot Analysis

Tissue homogenates were prepared by placing left ventricle tissue in ice-cold RIPA lysis buffer (pH 7.0) from Santa Cruz Biotechnology (Santa Cruz, CA; SC-24948) containing protease inhibitors and phosphatase inhibitors. About 40 µg homogenates were analyzed using Western blot. The primary antibodies used in western blot analyses were: Rabbit anti-CD31, 1:500 dilution (Abcam Inc., Cat. No.: ab28364); Rabbit anti-α-smooth muscle actin, 1:500 dilution (Abcam Inc., Cat. No.: ab5694); Rabbit anti-GAPDH antibody, 1:1000 dilution (Santa Cruz Inc., Cat. No.: sc-25778). Secondary antibodies included goat anti-rabbit IgG-HRP (Santa Cruz Inc., Cat. No.: sc-2030); goat anti-mouse IgG-HRP (Santa Cruz Inc., Cat. No.: sc-2031). The Western blot images were cropped for presentation in each figure, and the uncropped Western blot images were provided in the Supplementary Info File.

### Masson’s Trichrome Staining for Fibrosis

Left ventricle sections fixed in 4% formaldehyde buffer solution (pH 7.2) were paraffin-embedded and cut into a thickness of 4 μm onto a microscopy slide. The tissue sections were deparaffinized with xylene and rehydrated by sequential incubations in ethanol and water followed by Masson’s Trichrome staining. The whole-slide scanning was performed on these slides using an Olympus whole-slide scanning system. Cardiac fibrosis was calculated and presented as percentage area of blue staining versus red staining using a computer aided morphometry as previously described^37,38^.

### Immuno-Fluorescent Staining of CD31 and CD34

Paraffinized heart tissue sections (4 µm in thickness) were steamed for 15 min in the Trilogy solution from Sigma-Aldrich (Cat. No. 920 P) for deparaffinization and antigen retrieval. Afterwards, the slides were blocked with 1% BSA in TBS-T solution followed by incubating overnight at 4 °C with anti-CD31 antibody from Abcam (Cat. No. ab28364, 1:50 dilution) or anti-CD34 antibody from R&D (Cat. No. AF6518). Alexa fluor 594 conjugated anti-rabbit antibody from ThermoFisher) (Cat. No. A-11012, 1;100 dilution) was used to visualize the CD31 immunostaining, and Alexa fluor 594 conjugated anti-sheep antibody from Abcam (Cat. No. ab96937) was used to visualize CD34. For co-immunostaining of CD31 and CD34, the secondary antibody for CD31 was switched to Alexa fluor 488 conjugated anti-rabbit antibody from Abcam (Cat. No. ab150073). Fluorescent images of CD31 and CD34 staining were taken using a Leica confocal microscope. In each slide, 5 images were randomly taken from different areas of the slide. Images were analyzed using FiJi ImageJ software to calculate the percentage of CD31 positive areas.

### Measurement of Cotinine

An ultra-high performance liquid chromatography-tandem mass spectrometry (UPLC-MS/MS) method was used to measure the plasma cotinine concentration as previously described^39^. Plasma samples were spiked with 0.05% aqueous formic acid containing a deuterated internal standard of cotinine, extracted with a solid phase resin, and analyzed using LC-MS/MS via multiple reaction monitoring (MRM) to quantitate cotinine. Identification of samples were blinded to the LC-MS experimenter. Briefly, 15 µl of plasma was transferred to a 1.5 mL microcentrifuge tube followed by addition of dH_2_O (200 µL, 0.05% formic acid [FA]) containing cotinine-*d*^3^ (250 nM), followed by dilution with ammonium acetate (10 mM, 200 µL). The mixture was applied to a biotage WCX solid phase extraction column. The column was washed successively with ammonium acetate (500 µL at 50 mM), isopropyl alcohol (125 µL) and dichloromethane (500 µL). Analytes were eluted with a mixture of dH_2_O:isopropyl alcohol (85:15) +0.1% FA (125 µL) followed by LC-MS analysis via a Shimadzu Nexera XR UPLC coupled with a Shimadzu 8050 triple quadrupole mass spectrometer. The instrument was optimized for MRM transitions using analytical grade standards of cotinine and cotinine-*d*^3^, (Sigma Aldrich and Cambridge Isotopes). The total ion chromatograms (TIC) for each MRM event were integrated to determine the area under the curve (AUC). Quantitation was carried out using a calibration curve.

### ELISA Measurement of plasma VEGF

The Plasma VEGF was measured using a commercial available ELISA kit from R&D System (Cat. No. MMV00). Plasma samples were first diluted 5 times with diluent buffer provided from the kit, then a 50 µL of the diluted samples or the serially diluted standards of VEGF were added to each well of a 96-well plate. The antibody incubation and washing procedures were performed following the manufacturer’s protocol. Plasma concentration of VEGF was quantified against the estamblmished standard curve.

### Statistical Analyses

Data are presented as Mean ± Standard Error of the Mean (SEM). Data were analyzed using Student t-test or One-way ANOVA where appropriate.

## Supplementary information

Supplementary Info File

## Data Availability

All available data is included in this manuscript.
