# Supplementary material for: The Effect of Electronic-Cigarette Vaping on Cardiac Function and Angiogenesis in Mice
Source: Sci Rep. 2019 Mar 11;9:4085. doi: 10.1038/s41598-019-40847-5 (PMC6411855; doi:10.1038/s41598-019-40847-5)
Supplement: Supplementary file 1 — Supplementary Info File [file 41598_2019_40847_MOESM1_ESM.pdf]

The Effect of Electronic-Cigarette Vaping on Cardiac Function and Angiogenesis in Mice

Huilin Shi[1*], Xiaoming Fan[1*], Austin Horton[2], Steven T. Haller[1], David J. Kennedy[1], Isaac T. Schiefer[2], Lance Dworkin[1], Christopher J. Cooper[1], Jiang Tian[1&]

[1]Department of Medicine, University of Toledo College of Medicine, Toledo, OH 43614

[2]Department of Medicinal and Biological Chemistry, College of Pharmacy and Pharmaceutical Sciences, University of Toledo, Toledo, OH 43614

***** These authors contribute equally to the manuscript.

Short Title: E-cigarette Vaping and Angiogenesis

[&] To whom correspondence should be addressed:

Jiang Tian, Ph.D.

Associate Professor

Department of Medicine

University of Toledo

3000 Arlington Avenue

Toledo, OH

Tel.: (419) 383-3510

E-mail: Jiang.Tian@utoledo.edu

Supp_Fig. 5A

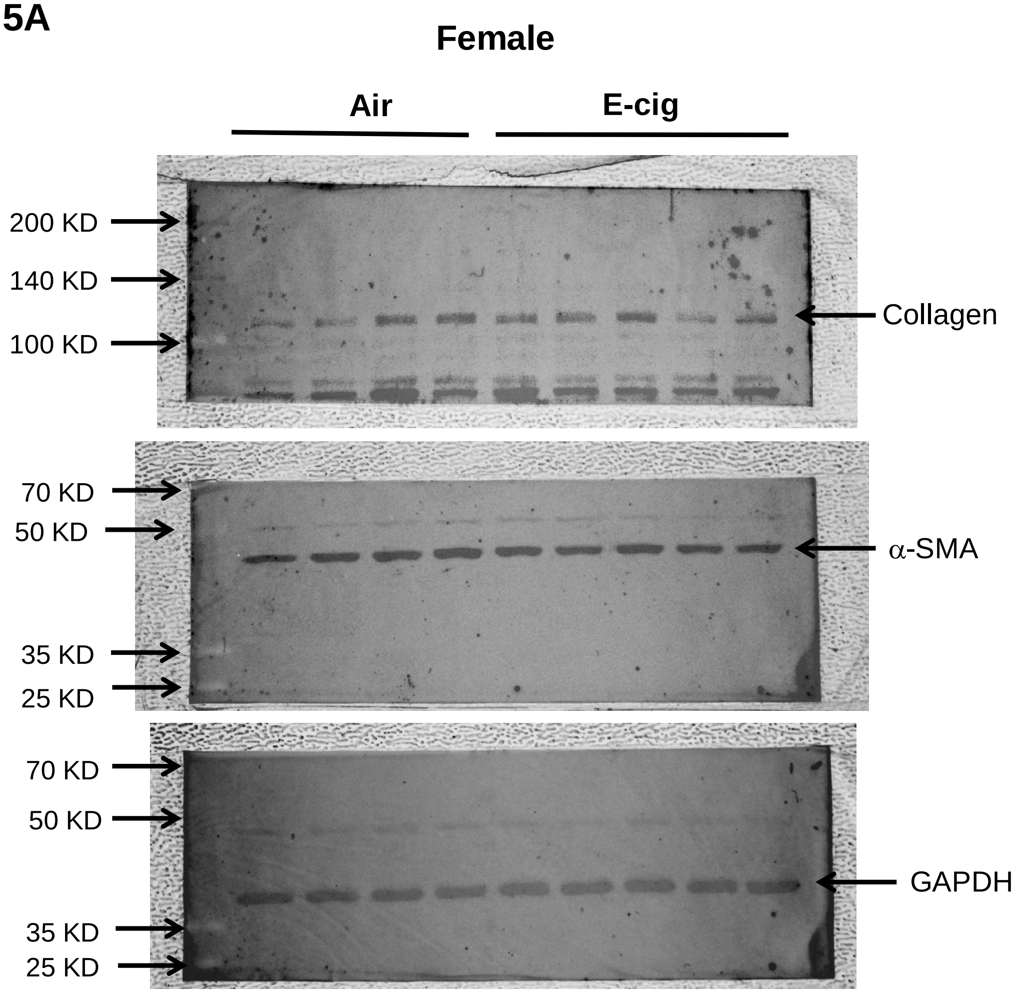

Female
Air
E-cig
200 KD
140 KD
100 KD
Collagen
70 KD
50 KD
α-SMA
35 KD
25 KD
70 KD
50 KD
GAPDH
35 KD
25 KD

Supp_Fig. 5B

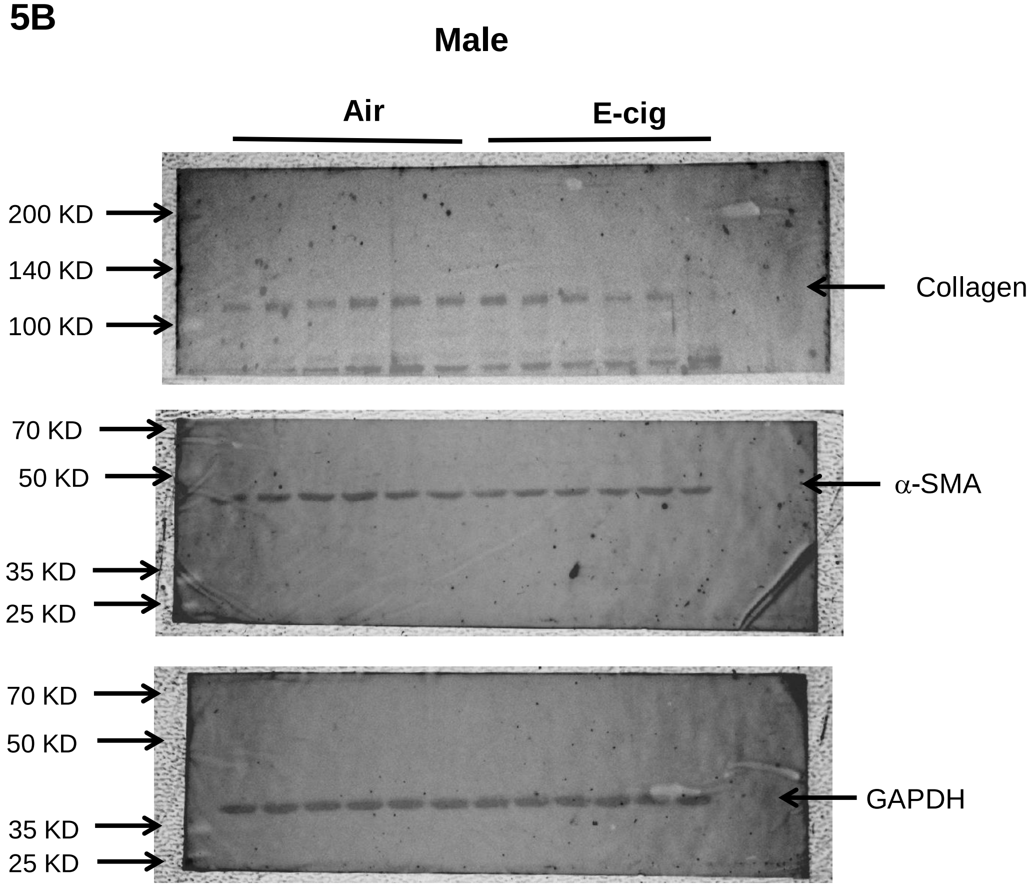
Male
Air
E-cig
200 KD
140 KD
100 KD
Collagen
70 KD
50 KD
35 KD
25 KD
α-SMA
70 KD
50 KD
35 KD
25 KD
GAPDH

Supp_Fig. 9

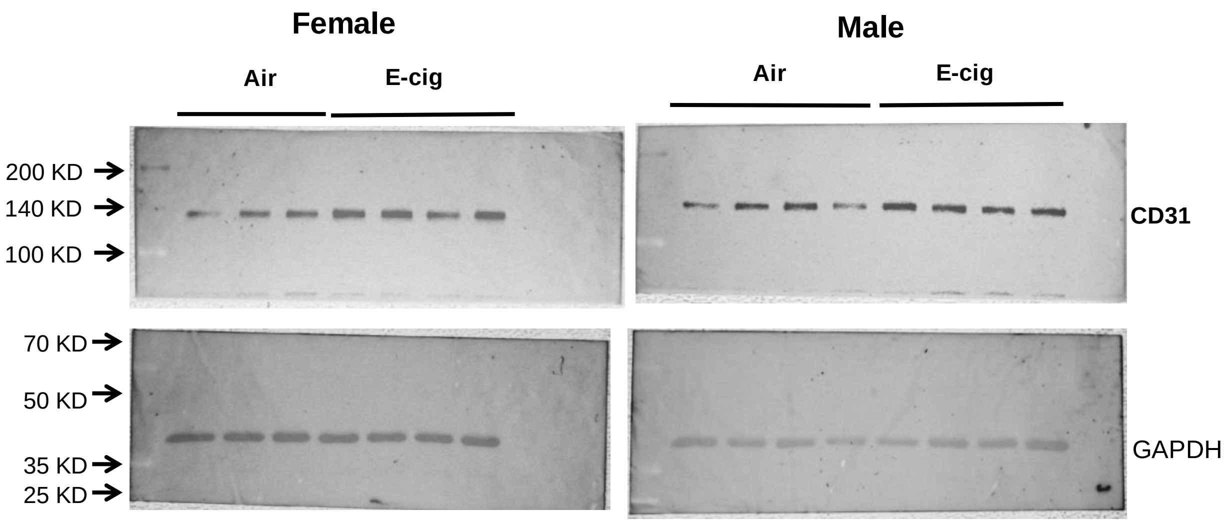
Female
Male
Air
E-cig
Air
E-cig
200 KD
140 KD
100 KD
CD31
70 KD
50 KD
35 KD
25 KD
GAPDH
